# Experimental Mouse Models of Disseminated Candida auris Infection

**DOI:** 10.1128/mSphere.00339-19

**Published:** 2019-09-04

**Authors:** Hong Xin, Farhan Mohiuddin, Jensen Tran, Abby Adams, Karen Eberle

**Affiliations:** a LSU Health Sciences Center New Orleans, New Orleans, Louisiana, USA; b Loyola University New Orleans, New Orleans, Louisiana, USA; Carnegie Mellon University

**Keywords:** disseminated candidiasis, *Candida auris*, neutrophil elastase (NE), complement, *Candida*, candidiasis, immunocompromised hosts, mouse models

## Abstract

Disseminated candidiasis is a life-threatening disease and remains the most common bloodstream infection in hospitalized patients in the United States. Despite the availability of modern antifungal therapy, crude mortality in the last decade has remained unacceptably high. In particular, Candida auris is a multidrug-resistant, health care-associated fungal pathogen and has recently emerged as the first fungal pathogen to cause a global public health threat. A reliable animal model for disseminated C. auris candidiasis is therefore needed to study the unique aspects of this little-known host-pathogen interaction. In this study, we established an inbred A/J intravenous model as an appropriate model for human disseminated C. auris infection. We found that C5 deficiency in A/J mice results in a complex phenotype characterized by rapid fungal proliferation in target organs and the development of a unique and rapidly fatal response. In contrast, C57BL/6J mice and mice deficient in neutrophil elastase (NE^−/−^) survived high-dose C. auris intravenous challenge, even with cyclophosphamide (CY)-induced immunosuppression. Our study is the first to provide insight into the role of C5 in the host responses to C. auris invasive infection and establishes an inbred A/J mouse model of systemic C. auris infection without CY-induced immunosuppression.

**IMPORTANCE** In the last decade, Candida auris has emerged globally as a multidrug‐resistant fungal pathogen. Although C. auris was initially isolated from the external ear canal, it can cause outbreaks of invasive infections with very high mortality and comorbidities. Recent reports highlight the ongoing challenges due to organism misidentification, high rates of multifungal drug resistance, and unacceptably high patient mortality. The assessment of C. auris virulence in a specific genetic deficiency mouse model of invasive C. auris infection in this study contributes to the little knowledge of host defense to C. auris infection, which holds promise as a model for investigating the pathogenesis of C. auris invasive infection, exploring the immune responses elicited by the fungus, evaluating the possible induction of immunity to the infection, and targeting candidates for an antifungal vaccine.

## Abstract (precis)

In the last decade, Candida auris has emerged globally as a multidrug‐resistant fungal pathogen. Although C. auris was initially isolated from the external ear canal, it can cause outbreaks of invasive infections with very high mortality and comorbidities. Recent reports highlight the ongoing challenges due to organism misidentification, high rates of multifungal drug resistance, and unacceptably high patient mortality. The assessment of C. auris virulence in a specific genetic deficiency mouse model of invasive C. auris infection in this study contributes to the little knowledge of host defense to C. auris infection, which holds promise as a model for investigating the pathogenesis of C. auris invasive infection, exploring the immune responses elicited by the fungus, evaluating the possible induction of immunity to the infection, and targeting candidates for an antifungal vaccine.

## INTRODUCTION

Since 2009, Candida auris has been an emerging fungus that presents a serious global health threat. This novel Candida species was initially isolated from the external ear canal of a patient in Japan. This fungus causes deep‐seated invasive infections with a very high death rate and causes mainly bloodstream, wound, and ear infections (1, 2). Indeed, C. auris is the very first fungal pathogen to cause a global public health threat (3).

Little is known about the virulence mechanisms that lead to the high transmissibility of C. auris to cause invasive disease following introduction into the human skin or gastrointestinal (GI) tract. Understanding host defense mechanisms and fungal virulence is critical for developing new strategies to prevent and control the invasive infection due to this multiresistant pathogen. The laboratory mouse is an acknowledged model that recapitulates human disease with remarkable fidelity for the study of disseminated candidiasis (4), In particular, intravenous infection with Candida albicans blastospores causes a pathology that closely resembles the human condition and is characterized by active fungal replication in the kidney, brain, and heart, with death usually resulting from kidney failure (5). The murine intravenous model of disseminated C. albicans infection is well characterized and reproducible, and current knowledge of immunity to *Candida* spp. has been gleaned almost exclusively from studies on C. albicans, the most common disease-causing species.

To date, there are several published animal models for C. auris systemic infection (6), such as mammals (7–10) and the larvae of the moth Galleria mellonella (10). In order to mimic the human disease that is normally caused by penetration of physical barriers, a mammalian animal would be most rational. For example, the Fakhim group reported a comparative study of *Candida* sp. virulence using an immunocompetent outbred ICR mouse model of systemic infection. They showed the high virulence of C. albicans isolates, which caused 80% mortality in infected mice, followed by C. auris, with which infected ICR mice exhibited 30 to 40% survival until the end of the experiment (7). In another study, the virulence of a single C. auris isolate obtained from a Chinese fungemic patient was evaluated in an intravenous BALB/c mouse model of *Candida* disseminated infection. BALB/c mice infected with 1 × 10^6^ cells of C. albicans (SC5314) via the tail vein died within 6 days, and mice infected with 1 × 10^7^ cells of C. auris showed 100% survival during the same period (9). In a study by Ben-Ami et al., one C. auris strain, TA005-14, was used to lethally infect BALB/c mice, and 20% survival was observed; they also observed distinct yeast cell aggregates in the kidneys of mice, which suggests that aggregation might be a mode of immune evasion and persistence in tissue (8). With regard to the pathogenicity of C. auris, aggregate-forming isolates exhibit significantly less pathogenicity than do their nonaggregating counterparts; however, Ben-Ami’s group did not clearly state what the cell phenotype of this isolate inoculum was when used for lethal challenge. Indeed, a reliable animal model for disseminated C. auris infection is needed to study the unique aspects of this host-pathogen interaction. Due to the scarcity of reliable animal models of disseminated C. auris infection, as well as the lack of clinical and experimental studies of C. auris pathogenicity, the aim of this study was to evaluate the virulence of C. auris in both immunocompetent and cyclophosphamide (CY)-induced immunosuppressed inbred murine models of disseminated infection.

Neutrophils are critical for the control of numerous invasive fungal infections, including candidiasis. Neutropenia is one of the most common risk factors for the development of hematogenously disseminated candidiasis in humans (11, 12), in which the mortality is above 50% despite antifungal therapy (13–16). Recently, the Nett group reported that evasion of neutrophil attack explains the virulence of C. auris (3). Unlike many fungal infections, neutropenia has not been reported as a common risk factor for C. auris infection (17, 18), which prompted us to explore if the interaction between neutrophils and C. auris accounts for the unexplained virulence of this emerging pathogen. Neutrophil elastase (NE) is an important component of neutrophil extracellular traps (NET) with a role in immunity against fungal infection, and NE has been implicated in the host defense against microbial or fungal pathogens using NE-deficient (NE^−/−^) mice (19–22). Despite its limited virulence compared to that of C. albicans, *in vitro* assays conducted with human neutrophils showed that C. auris is less effective than C. albicans in triggering neutrophil engulfment and NET production (6). To clarify the contribution of NE in the defense against C. auris, we first evaluated the virulence of two C. auris isolates in NE-deficient mice (B6.129 × 1-Elanetm1Sds/J, referred to as NE^−/−^) and wild-type (WT) C57BL/6 mice. AR-0381 is a drug-susceptible East Asian isolate, and AR-0386 is an azole-resistant (Erg11 Y132F) South American one. In a preliminary study, we used both strains to infect BALB/c mice by intravenous challenge with inocula ranging from 5 × 10^5^ to 5 × 10^7^ cells without achieving mortality. The C57BL/6 background strain by genetic control is more resistant to C. albicans systemic infection than is the BALB/c strain. In this study, the survival of both the C57BL/6 and NE^−/−^ strains after high-dose infection was unimpaired, suggesting that NE is not essential for survival. The greater susceptibility of A/J mice, a C5-deficient inbred strain, to systemic challenge with C. albicans and other non-*albicans Candida* (NAC) species (23) prompted us to investigate whether A/J mice are more susceptible to C. auris disseminated infection. Here, we conducted a comparative study of the survival and organ CFU of invasive C. auris infection in C57BL/6, NE^−/−^, and A/J mice. We found that C5 deficiency in A/J mice results in a phenotype characterized by rapid fungal proliferation in target organs and the development of a unique and rapidly fatal (within 24 to 48 h) response. In contrast, C57BL/6J and NE^−/−^ mice survived high-dose C. auris challenge even with immunosuppressant (CY) treatment.

## RESULTS

### Comparison of C. albicans and C. auris virulence in NE^−/−^ and C57BL/6 mice.

**FIG 1:**
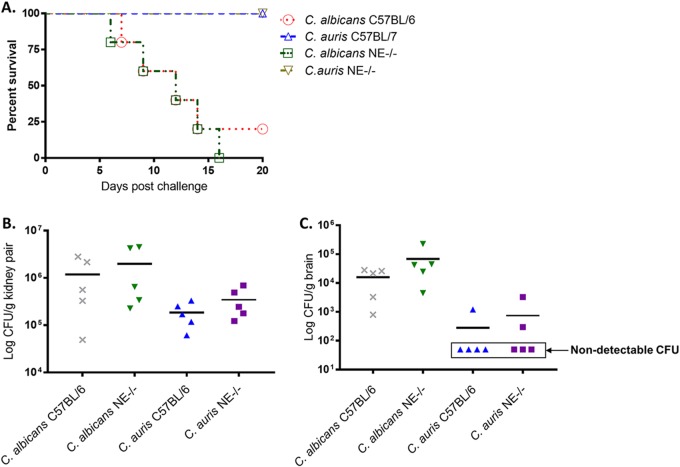
Comparison of virulence of C. albicans and C. auris in neutrophil elastase-deficient (NE^−/−^) mice and WT C57BL/6 mice. (A) NE^−/−^ and C57BL/6J female 8-week-old mice were infected i.v. with 5 × 10^5^ C. albicans SC5314 cells or 2 × 10^8^ C. auris 0386 yeast cells. Mice were observed twice daily for 20 days for clinical signs of illness or mortality. The results are representative of five mice per inoculum. Survival times were statistically evaluated by the Kaplan-Meier statistic (GraphPad Prism, version 7), and *P* values less than 0.05 were considered statistically significant. Mice challenged with C. auris AR-0386 survived significantly longer than did control mice that received C. albicans SC5314 (*P = *0.0023 for NE^−/−^ and *P = *0.0031 for C57BL/6). (B and C) In NE^−/−^ mice, the fungal burdens of C. albicans and C. auris in targeted organs, the kidneys (B) and brain (C), are higher than those in wild-type C57BL/6 mice (*P* < 0.05); however, there is no significant difference between the two mouse strains (*P* = 0.25). Fungal burden in the brains of both strains remained low after C. auris challenge. Only 1 out 5 mice in WT C57BL/6 and 2 out 5 in NE^−/−^ mice had detectable CFU in the brain. All fungal burden data were analyzed by a multivariate analysis of variance (MANOVA).

As NE is an important serine protease implicated in antifungal immune responses (3), we first evaluated the virulence of C. auris in NE^−/−^ and its wild-type C57BL/6 mice. Initial experiments were conducted to challenge mice of both strains with high doses of C. auris intravenously (i.v.). A lethal dose of C. albicans SC 5314 (5 × 10^5^ cells) was used as a positive control to induce acute invasive candidiasis, as well to provide a virulent comparison to C. auris. Mice were inoculated i.v. with C. auris (2 × 10^8^ cells) and observed twice daily for 20 days for morbidity and mortality. As shown in Fig. 1, all mice inoculated with C. auris survived and appeared to be clinically normal. In contrast, systemic infection with C. albicans (5 × 10^5^ cells) resulted in 80 to 100% mortality by 15 days postinfection, which was associated with clinical signs of disease, including weight loss, lethargy, and a ruffled appearance. It is important to note that in NE^−/−^ mice, the fungal burden of C. auris in both targeted organs, the kidneys and brain, is higher than that in wild-type C57BL/6 mice. These results show that while NE may contribute to antifungal immunity, it is not required for survival. We tested two clinical isolates of C. auris (AR-0381 and AR-0386) for their lethality in mice. Neither strain showed mortality in mice.

### Comparison of virulence of C. auris in CY-induced immunosuppressed C57BL and NE^−/−^ mice.

**FIG 2:**
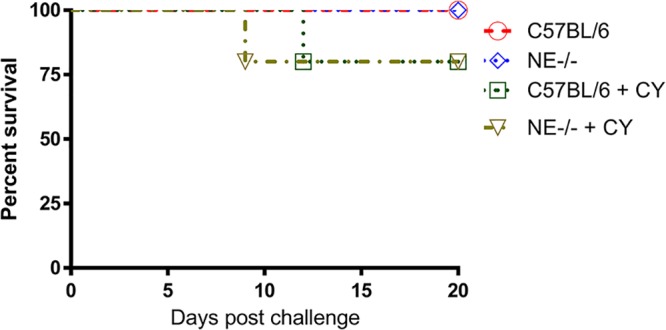
Immunosuppressed intravenous mouse models of C. auris disseminated infection. C57BL/6 and NE^−/−^ mice received a 200-mg/kg dose of cyclophosphamide (CY) i.p. on day −3. Prolonged neutropenia was maintained by giving each animal a 150-mg/kg dose of CY i.p. every 7 days after infection. On day 0, mice were intravenously infected with 2 × 10^8^ viable C. auris cells in 0.1 ml DPBS. As controls, mice without CY treatment were challenged with the same dose of C. auris cells at the same time. There is no significant difference in the survival of the two strains with and without CY-induced immunosuppression (*P* = 0.45). Survival times were statistically evaluated by the Kaplan-Meier statistic (GraphPad Prism, version 7), and *P* values less than 0.05 were considered statistically significant.

Immunocompromised patients are the highest-risk group for acquiring C. auris systemic infection. With the aim of mimicking the clinical situations in humans, experimental invasive C. auris infections were induced in both C57BL/6 and NE^−/−^ mice rendered immunocompromised by pretreatment with cyclophosphamide (24–26). We evaluated two different clinical isolates of C. auris (AR-0381 and AR-0386) for their lethality in a CY-treated immunosuppressed mouse model of both strains with different doses ranging from 2 × 10^7^ to 2 × 10^8^ cells; only the high-intravenous-inoculum 2 × 10^8^ cells/mouse dose of C. auris AR-0386 showed 20% mortality in the mice. Therefore, in the following experiments, the AR-3860 strain was our focus and was used for virulence testing in different mouse strains of a genetic background. The survival data show that CY-induced immunosuppressed mice of both strains are more susceptible to C. auris AR-0386; however, they remain resistant against systemic infection by C. auris, with only 20% mortality observed by 20 days postinfection (Fig. 2). Importantly, extending the observation period to 30 days postinfection did not change the percent mortality.

### Evaluation of C. auris virulence in naive and CY-treated immunosuppressed A/J mice.

**FIG 3:**
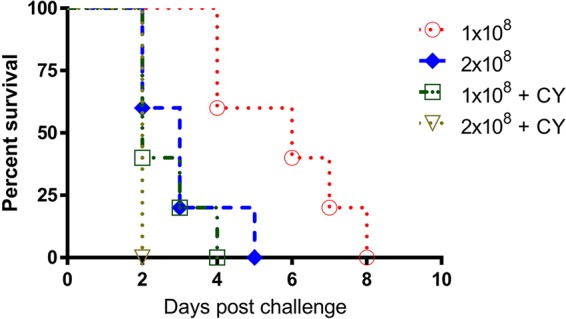
Evaluation of C. auris virulence in a naive and immunosuppressed A/J mouse model of disseminated candidiasis. Female 8-week-old A/J mice received a 200-mg/kg dose of cyclophosphamide (CY) i.p on day −3. Prolonged neutropenia was maintained by giving each animal a 150-mg/kg dose of CY i.p. every 7 days after infection. On day 0, mice were infected intravenously with 1 × 10^8^ or 2 × 10^8^ viable C. auris cells in 0.1 ml DPBS. As controls, age- and sex-matched A/J mice without CY treatment were challenged with the same two inoculum sizes of C. auris cells at the same time. There is no significant difference in survival between different doses or between the groups immunosuppressed with CY or without CY-induced immunosuppression. Survival times were statistically evaluated by the Kaplan-Meier statistic (GraphPad Prism, version 7), and *P* values less than 0.05 were considered statistically significant.

Analyses of the pathophysiology and host responses to acute C. albicans and to NAC infection, as demonstrated by our group and others, show that A/J mice are extremely sensitive to invasive *Candida* infection (27–29). The increased susceptibility of A/J mice to systemic challenge with *Candida* prompted us to investigate whether A/J mice are more susceptible to C. auris disseminated infection. To do this, we first tested two inoculum sizes of a C. auris isolate in both naive and CY-treated A/J mice. Compared to C57BL/6 and NE^−/−^, both naive and CY-treated immunosuppressed A/J mice are much more susceptible to C. auris. After intravenous challenge with half the inoculum of organisms (1 × 10^8^ cells) used in C57BL and NE^−/−^ mice, 100% mortality was observed in naive A/J mice within 8 days even without CY pretreatment (Fig. 3). Particularly, an intravenous dose of 2 × 10^8^ C. auris cells showed 100% mortality in A/J mice pretreated with CY within 48 h. With the same inoculum challenge and CY treatment, C57BL/6 and NE^−/−^ mice are resistant to C. auris bloodstream infection with 80 to 100% survival. After two independent experiments, we developed an naive inbred A/J mouse infection model by screening two different clinical isolates of C. auris (0381 and 0386) for their lethality in mice (data not shown). We used a CY-treated BALB/c i.v. model challenged with either strain and only achieved 20% mortality with 0381 and 40% with 0386. Additionally, 0381 strain has a poor growth rate that was lower than that of the 0386 strain. Due to the lower virulence of 0381 compared to that of 0386 in our previous study, we then focused on testing C. auris 0386 in all the challenge experiments in this study.

### Influence of CY-induced immunosuppression on fungal burdens in targeted organs during the course of C. auris invasive infection.

**FIG 4:**
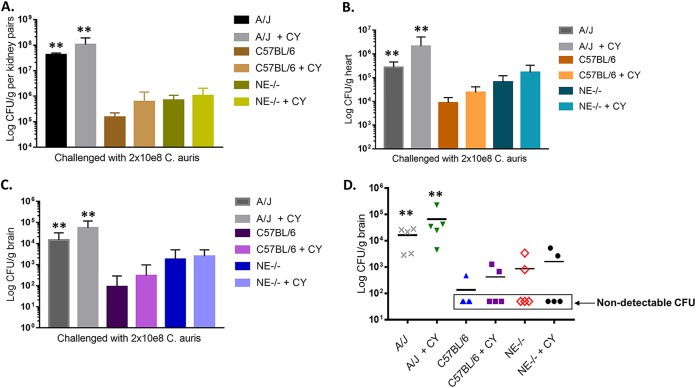
Influence of immunosuppression on fungal burdens in brain, heart, and kidneys in mouse models of systemic C. auris infection. (A to C) Three mouse strains, C57BL/6, NE^−/−^, and A/J mice, with and without CY pretreatment before C. auris 0386 intravenous challenge (2 × 10^8^ cells), were compared for fungal burdens in targeted organs, including kidney (A), brain (B), and heart (C). Data are expressed as the mean + standard deviation (S.D.) (*n* = 5). *, *P* = 0.05; **, *P* = 0.01 by two-tailed Student’s *t* test between the indicated A/J groups (with or without CY-induced immunosuppression) compared to the appropriate C57BL/6 (wild type) groups (with or without CY-induced immunosuppression). (D) Fungal burdens in brain of each mouse in each experimental group are shown. In both C57BL/6 and NE^−/−^ strains, only 1 or 2 out of 5 mice for each group had detectable fungal CFU, independent of CY-induced immunosuppression. A/J mice, with or without CY treatment, had significant fungal burdens in brain compared to C57BL/6 mice.

Unlike C. albicans, C. auris does not induce a lethal phenotype in WT C57BL/6 and NE^−/−^ mice, even with infections of high inoculum. In this study, we compared the effect of CY-induced immunosuppression on the outcome of C. auris systemic infection in NE^−/−^, WT C57BL/6, and A/J mice. We infected mice with or without CY pretreatment and terminated the experiment at 48 h postinfection. The CFU counts in the organs of cyclophosphamide-treated mice of C57BL/6 and NE^−/−^ mice were higher than those in mice without CY treatment. C5-deficient A/J mice treated with CY had the most CFU in all targeted organs compared to other groups (Fig. 4A to C). Naive A/J mice always had higher CFU counts than did CY-treated C57BL/6 and NE^−/−^ mice, and this was independent of host CY-induced immunosuppression. In particular, A/J mice had significantly higher (*P* < 0.01) numbers of yeast in the brain than did C5-sufficient strains, where only 1 or 2 out of 5 in the C57BL/6 (with and without CY treatment) groups had detectable CFU and 2 out of 5 in NE^−/−^ groups had detectable CFU in the brain. However, all A/J mice, regardless of the CY-induced immunosuppression status, had high fungal burden in the brain (Fig. 4D).

## DISCUSSION

A reliable animal model for disseminated C. auris infection is critical for investigating the unique aspects of this little-known host-pathogen interaction. First, to determine the contribution of the antimicrobial molecule neutrophil elastase (NE) in combating C. auris invasive infection, we used mice with deficient NE (NE^−/−^) and their wild-type control C57BL/6 mice challenged with high doses of C. auris. Interestingly, the survival of both groups after infection was unimpaired, suggesting that NE is not essential for survival here. However, NE^−/−^ mice had a higher fungal burden in all the targeted organs than did wild-type controls, indicating a partial contribution of NE to fungal immunity. Consistent with reports by other groups, our study also showed a higher pathogenicity for C. albicans over C. auris. Most groups asserted that C. auris could not effectively infect and disseminate in mice unless they were immunocompromised (2). In our study, a larger C. auris inoculum size (2 × 10^8^ yeast cells/animal) was administered compared to the 3 × 10^7^ dose used by others, while both C57BL/6J and NE^−/−^ strains remained resistant, as measured by survival following intravenous injection of C. auris blastospores.

With the aim of mimicking the clinical situations in humans with cancer chemotherapy, experimental systemic C. auris infections were also induced in C57BL/6 and NE^−/−^ mice rendered immunocompromised by pretreatment with cyclophosphamide, the most commonly used immunosuppressant for immunosuppressed animal models. The immunosuppressed mice were more susceptible, with increased fungal burdens in targeted organs; however, they survived and tolerated a higher kidney fungal burden without clearing the infection, where 80% survival in both strains was observed by 20 days postinfection. Fungal burden increased in targeted organs after C57BL/6 and NE^−/−^ mice treated with CY; however, the increased fungal replication did not dramatically affect survival. Therefore, an assessment of susceptibility to C. auris using intravenous C57BL/6 and NE^−/−^ mouse models has been limited to CFU in targeted organs and interference with immunosuppressant drugs. Since the inbred A/J mouse strain is exquisitely sensitive to infection with C. albicans, and since we have successfully established the A/J CY-treated immunosuppressed mouse model for Candida glabrata disseminated candidiasis (23), we then developed a hematogenous model of C. auris infection in naive inbred A/J mice to study the *in vivo* virulence of C. auris that may lead to death without CY-induced immunosuppression. Our results showed that A/J mice were significantly more susceptible than were the other two mouse strains to C. auris and showed outcomes of a typical systemic infection of candidiasis. An intravenous 1 × 10^8^ inoculum size of C. auris already showed 100% mortality within 8 days in A/J inbred mice without immunosuppressant treatment. Additionally, CY-treated A/J mice died rapidly within 48 h. Therefore, the A/J naive mouse model can be considered a valuable model of human disseminated C. auris infection. Importantly, this study represents the first demonstration of this relationship of both NE^−/−^ and A/J mice with C. auris.

In our preliminary study, we investigated the differential susceptibilities of inbred A/J, BALB/c, and C57BL/6J (B6) strains to acute infection with C. albicans (23). The A/J mouse strain is exquisitely sensitive to candidiasis, there are high fungal loads in target tissues, and rapid death occurs within 48 h following intravenous injection of 5 × 10^5^ cells/0.1 ml C. albicans SC5314 blastospores. On the other hand, BALB/c mice survive up to 1 to 2 weeks following the same inoculum by i.v. infection and ultimately die of kidney failure; C57BL/6 mice were more resistant and died within 2 to 3 weeks with the inoculum of 1 × 10^6^ cells/0.1 ml. The Mullick group reported that in A/J mice, tissue damage is associated with increased replication of *Candida* spp. at all targeted organs, with the kidney having the highest fungal burden. However, histological and biochemical analyses showed that it is the heart that suffers the worst damage, which is due to significant leukocyte infiltration in the vicinity of *Candida* hyphae in the heart. Cell infiltration then resulted in strong proinflammatory activity by activating many cytokines and chemokines. Therefore, the heart is the organ that is most affected, and it ultimately fails in C5-deficient mice (30). That is the case with C. albicans; however, we did not know if it is the same with C. auris. We have evaluated three cytokines, tumor necrosis factor alpha (TNF-α), interleukin-6 (IL-6), and monocyte chemotactic protein 1 (MCP-1), in serum samples from A/J mice 24 to 48 h postchallenge by the use of cytokine enzyme-linked immunosorbent assay (ELISA) kits from BD Biosciences. There were no significantly elevated levels of any cytokine in the sera of A/J mice following infection.

Furthermore, in mouse models of *Candida* invasive infection, susceptibility is under a complex genetic control that affects the onset of infection, type and severity of disease developed, and associated pathologies. Inbred mouse strains vary widely in their degree of innate susceptibility to systemic candidiasis, being either highly susceptible (A/J and DBA/2) or highly resistant (C57BL/6J). Due to genetic resistance, we have tried but failed to establish acute systemic candidiasis with 80 to 100% mortality in BALB/c, C57, or NE^−/−^ mice. Therefore, by use of a highly susceptible A/J strain for a CY-induced immunosuppressed infection model, we surprisingly established a naive A/J model for C. auris invasive infection. This systemic infection without CY-induced immunosuppression that leads to acute invasive candidiasis is more suitable for the evaluation of immune responses or comparison of the pathogenicity of C. albicans strains without the interference of the consequences of immunosuppression.

As opposed to the C57BL/6J and NE^−/−^ mouse strains, the A/J mouse strain carries a loss-of-function mutation in the structural gene for the C5 component of the complement pathway (26, 27). Studies by our group show that C5 deficiency imparts susceptibility to NAC infections in the A/J strain (23), which further showed extreme susceptibility to C. auris invasive infection in this study. C5 plays several critical roles in the host response to infection, including target lysis and phagocyte recruitment. The central role of anaphylatoxin C5a in augmenting host proinflammatory cytokine production upon contact with C. albicans has been reported (29, 31), which may indicate a similar role for the complement system in the anti-C. auris host defense in mice in this study. The greater susceptibility of A/J mice to C. auris disseminated infection might also be associated with an exaggerated inflammatory response (23), indicating that the balance between pro- and anti-inflammatory mediators is crucial for host survival.

Taking together the data provided by us and other groups by the use of experimental mouse models, we conclude that C. auris exhibits less virulence than do C. albicans isolates. Nevertheless, C. auris is a multidrug-resistant fungus rapidly spreading worldwide as an agent of hospital-associated infections with cruel morbidity and mortality. Assessment of C. auris virulence in the A/J mouse strain in this study contributes to the knowledge of the host defense to C. auris infection. We have used A/J mice lacking C5 to examine its role in antifungal immunity and have shown that this complement component is essential for resistance to systemic infections with C. auris. Therefore, the A/J mouse holds promise as a model for investigating the pathogenesis of C. auris invasive infection, exploring the immune responses elicited by the fungus, evaluating the possible induction of immunity to the infection, and targeting candidates for antifungal vaccines.

## MATERIALS AND METHODS

### *Candida* strains and culture conditions.

Three isolates of *Candida* were used to induce systemic infection in mice. C. albicans SC5314 (ATCC MYA-2876), C. auris AR-0381 (CDC), and C. auris AR-0386 (CDC) were grown as stationary-phase (24-h) yeast cells in glucose-yeast extract-peptone (GYEP; 2% glucose, 1% peptone, 0.3% yeast extract) broth at 37°C. *Candida* yeast cells were washed and suspended to the appropriate cell concentration (5 × 10^6^/ml, 1 × 10^9^/ml, or 2 × 10^9^/ml) in Dulbecco’s phosphate-buffered saline (DPBS; Sigma) and used to infect mice intravenously (i.v.), as described previously (32, 33).

### Mice.

Neutrophil elastase-deficient (NE^−/−^) mice on a C57BL/6 background (B6.129X1-*Elane^tm1Sds^*/J, 006112) and female 8-week-old WT C57BL/6J (000664) mice were obtained from JAX. A/J female 8-week-old mice (National Cancer Institute Animal Production Program, Frederick, MD) were used throughout. Mice were maintained in our AAALAC-certified animal facility, and all animal experiments were performed using a protocol approved by the Institutional Animal Care and Use Committee (IACUC) at the LSU Health Sciences Center (LSUHSC).

### Immunosuppression.

For CY-induced immunosuppressed mouse models, before challenge, C57BL/6, NE^−/−^, and A/J mice were immunosuppressed by intraperitoneal (i.p.) injection of a 200-mg/kg dose of cyclophosphamide (CY; Sigma-Aldrich) at day −3. The i.p. injection (150 mg/kg) was repeated every 7 days after infection (i.e., at days 7, 14, 21, and 28 postinfection) in order to maintain leukocytopenia and low neutrophil counts for the entire experimental procedure. This regimen has been shown to render mice neutropenic (absolute neutrophil count, <500 cells/mm^3^) within 3 to 4 days of the first cyclophosphamide dose, and neutropenia lasted until the termination of the experiments (up to day 30) (4, 23, 24). Total leukocyte and differential cell counts were determined on a hemacytometer and by Wright-Giemsa staining. Leukocyte and differential counts from peripheral blood were monitored every other day to verify the induction of leukocytopenia. The body weights of the mice were also measured daily and compared during the entire experimental period after the first CY or DPBS injection.

### Fungal challenge dose and assessment of protection.

Immunocompetent and CY-treated immunosuppressed mice and age- and sex-matched normal control mice (*n *=* *5 in each group) were injected intravenously with different doses of live C. auris yeast cells (1 × 10^8^, and 2 × 10^8^ in 0.1 ml DPBS) and C. albicans yeast cells (5 × 10^5^ in 0.1 ml DPBS). The challenged mice were monitored for the development of a moribund state, defined as being listless, disinterested in food or water, and nonreactive to finger probing. At that time, each mouse was sacrificed, and kidneys, hearts, and brains were homogenized in DPBS and plated onto nutrient agar to determine the CFU. All experiments were performed in duplicate. Tissue burden and mortality rate were used as virulence markers.

### Statistical analysis.

Survival times were statistically evaluated by the Kaplan-Meier statistic (GraphPad Prism, version 7), and statistical significance was subsequently calculated for each preset time point of analysis. All fungal burden data were analyzed by a multivariate analysis of variance (MANOVA). This multivariate analysis allowed for correlations of the CFU obtained from organs within the same animal to be determined. All statistical tests were performed at a 5% significance level.
